# Sbno1 mediates cell–cell communication between neural stem cells and microglia through small extracellular vesicles

**DOI:** 10.1186/s13578-024-01296-4

**Published:** 2024-09-29

**Authors:** Yifan Zhang, Zhihan Zhu, Zhinuo Li, Jia Feng, Jun Long, Yushu Deng, Waqas Ahmed, Ahsan Ali Khan, Shiying Huang, Qingling Fu, Lukui Chen

**Affiliations:** 1 Department of Neurosurgery, Southern Medical University Hospital of Integrated Traditional Chinese and Western Medicine, Southern Medical University, Guangzhou, China; 2 Department of Neurology, Zhongda Hospital Southeast University, Nanjing, China; 3 Department of Neurosurgery, The Aga Khan University, Karachi, Pakistan; 4 Otorhinolaryngology Hospital, The First Affiliated Hospital, Sun Yat-sen University, Guangzhou, China

**Keywords:** Sbno1, NSCs, Ischemic stroke, Neural repair, Neuroinflammation

## Abstract

**Background:**

Neural stem cells (NSCs) play a crucial role in the progress of ischemic stroke. Research on zebrafish embryonic demonstrates an association between Strawberry Notch 1 (Sbno1) and central nervous system development. However, the regulation and underlying mechanism of Sbno1 in NSCs have not been studied yet. Here, we investigated the role and the mechanism of Sbno1 in NSCs development and the potential therapeutic value of Sbno1 in ischemic stroke.

**Methods:**

Adeno-associated virus (AAV) was used for overexpression or knockdown of Sbno1 in vitro or in vivo. A mouse model of MCAO was established to evaluate the neuroprotective effects of AAV-Sbno1, including balance beam test, rotarod test, and strength evaluation. H&E and immunofluorescence assessed neuronal impairment. Western blot and RT-qPCR were used to detect the expression of Sbno1 and its downstream target genes. RNA-seq and western blot were performed to explore further molecular mechanisms by which Sbno1 promoted endogenous repair of NSCs and macrophages M2 polarization. CCK8 was conducted to assess the effects of Sbno1 on NSCs proliferation. The impact of Sbno1 on NSCs apoptosis was evaluated by flow cytometry. NSCs derived from small extracellular vesicles (sEV) were obtained using ultracentrifugation and identified through nanoparticle tracking analysis (NTA) and western blot analysis.

**Results:**

Our results showed that Sbno1 is highly expressed in the central nervous system, which plays a crucial role in regulating the proliferation of NSCs through the PI3k-Akt-GSK3β-Wnt/β-catenin signaling pathway. In addition, with overexpression of Sbno1 in the hippocampus, post-stroke behavioral scores were superior to the wild-type mice, and immunofluorescence staining revealed an increased number of newly generated neurons.

sEV released by NSCs overexpressing Sbno1 inhibited neuroinflammation, which mechanistically impaired the activation of the microglial NF-κB and MAPK signaling pathways.

**Conclusions:**

Our studies indicate that sbno1 promotes the proliferation of NSCs and enhances endogenous repairing through the PI3k-Akt-GSK3β-Wnt/β-catenin signaling pathway. Additionally, NSCs overexpressing sbno1 improve ischemic stroke recovery and inhibit neuroinflammation after ischemia by sEV through the MAPK and NF-κB signaling pathways.

**Supplementary Information:**

The online version contains supplementary material available at 10.1186/s13578-024-01296-4.

## Introduction

Stroke, along with coronary heart disease and cancer, is regarded as one of the three major diseases that significantly impact human health. Stroke encompasses both hemorrhagic and ischemic types, with ischemic stroke being the predominant form [1]. Thrombolytic therapy is an effective treatment for ischemic stroke [2]; however, its narrow time window restricts its accessibility to the majority of patients, leading to high mortality and disability rates associated with ischemic stroke [3, 4]. Even when patients receive timely thrombolytic treatment, the process itself can give rise to severe complications, notably cerebral hemorrhage [5].

NSCs possess the ability to differentiate into neurons, astrocytes, and oligodendrocytes [6]. For patients with post-ischemic stroke neurological impairments, NSCs therapy holds significant research value [7, 8]. Current studies on NSCs therapy for ischemic stroke involve the activation of endogenous NSCs in the hippocampal region for innate repair [9, 10], as well as the transplantation of exogenous NSCs through microsurgery [11]. Endogenous NSCs in the hippocampal region exhibit natural biological safety; however, their quantity is minimal and predominantly in a quiescent state [12, 13], resulting in a relatively restricted innate reparative capacity. Compared to the innate repair of NSCs, NSCs cultured in vitro maintain cellular stability, and the number of transplanted cells can be controlled [14]. However, exogenous transplantation presents inevitable challenges, such as immune rejection [15]. Even when NSCs derived from autologous induced pluripotent stem cells (iPSCs) are used to mitigate the risk of immune rejection [16], NSCs are delicate and exhibit poor tolerance. They require stringent in vitro cultivation conditions [17–19]. Following ischemic stroke, in addition to inadequate blood supply caused by vascular occlusion, the attack of neuroinflammation mediated by microglia further damages the cells in the ischemic penumbra, exacerbating the severity of the injury, expanding the extent of damage and reducing the survival rate of transplanted NSCs. This significantly impedes the therapeutic efficacy of NSCs.

Sbno1, a member of the Strawberry Notch family, remains relatively understudied in neuroscience. In the development of embryonic stem cells, embryos with suppressed Sbno1 expression fail to form blastocysts and undergo death [20]. Studies on mouse testicular development and spermatogonial stem cells have shown that knocking down the Sbno1 level results in the inactivation of the non-classical Wnt pathway, impacting the development of newborn mouse testes and spermatogonial stem cells [21]. Research on zebrafish embryonic development has demonstrated a clear association between Sbno1 and central nervous system development [22]. A clinical study has indicated a specific relationship between structural variations in Sbno1 and the occurrence of intellectual disabilities and related cognitive disorders such as autism and schizophrenia [23]. These studies indicate deeper connections between Sbno1 and the development of the central nervous system.

AAV does not integrate the target gene into the recipient cell’s genome, thus ensuring a high biological safety level. This study employs an AAV packaging system carrying Sbno1 for gene therapy, aiming to treat acute ischemic stroke. First, AAV-Sbno1 could activate and enhance quiescent endogenous NSCs’ proliferative capacity within the hippocampal region. This intervention is designed to augment the innate repair capabilities of NSCs while concurrently inhibiting the onset of neuroinflammation. The ultimate goal is to reduce further neural damage caused by neuroinflammation. In summary, our research studies the development of NSCs and the regulation of neuroinflammation based on the biological mechanisms of Sbno1. Our results explored the application of AAV-Sbno1 in gene therapy, offering a novel perspective on reducing the high disability rates associated with strokes and enhancing the quality of life for stroke patients.

## Materials and methods

### Animal

C57BL/6 mice were purchased from the Lab Animal Center of Southern Medical University (Guangzhou, China). All mice were housed in an environment with 50% humidity and 24 ℃ temperature, 12 h dark and light cycle, and received free diet and water access. All mice were maintained in the Lab Animal Center of Southern Medicine University under specific pathogen-free conditions. All animal experiment procedures were conducted according to a protocol approved by the Southern Medical University Ethics Committee.

### Isolation and culture of fetal NSCs

NSCs were obtained from mouse fetal brain tissue, as previously mentioned. Briefly, the lateral portion of the dorsal telencephalon (cortex) of embryonic day 13 (E13) C57/BL6 mice was isolated and disrupted with trypsin (5 min, 0.05% w/v) and then mechanically disrupted into single cells by repeated pipetting in medium DMEM/F12 (1:1) containing penicillin G (100 units/mL) and streptomycin (100 μg/mL). After centrifugation (5 min, 1,000 rpm), the pellet was resuspended in serum-free medium DMEM/F12 (1:1) supplemented with 2% B27 (Gibco, Grande Island, New York, USA), bFGF 20 ng/mL (Novoprotein Science Co., Ltd., Suzhou, China), EGF 20 ng/mL (Sino Biological Inc., Beijing, China), glutamine (2 mmol/L), 1% penicillin/streptomycin, and dissociated cells was cultured at a density of 5 × 10^4^ cells/mL. After 7 days, primary neurospheres were collected by low-speed centrifugation and dissociated into Single cells using chemical and mechanical methods. Then, the cells were re-inoculated to obtain new passages; all experiments were conducted using second-generation cells.

NSC differentiation was performed by plating 2.5 × 10^5^ or 5 × 10^4^ single cells on poly-D-lysine (PDL) (10 μg/mL)-coated 24 or 96 well plates, respectively, in a differentiation medium containing DMEM/F12 (1:1) supplemented with B27.

### Isolation of microglial

Primary microglials were prepared as previously described. Briefly, brains from postnatal 1- to 3-day-old mice were removed from the meninges, diced into 1 mm3 pieces, and incubated with 0.125% trypsin (Gibco, USA) with gentle shaking at 37 ℃ for 10 min. The dissociated cells were plated onto PDL-coated T-flasks filled with culture medium (DMEM/F12 containing 20% heat-inactivated fetal bovine serum [FBS], 1% penicillin, and 1% streptomycin). The cells were grown to confluence for 14 days. After 14 days of culture in vitro, the mature microglials were separated by shaking at 200 rpm for 2 h at room temperature.

### Cell culture and treatment

HEK293T cells (American Type Culture Collection number CRL-1653) were grown in DMEM supplemented with 10% FBS and incubated at 37 ℃ with 5% carbon dioxide.

Microglia were co-treated with After 24 h; the polarized microglia were cultured with LPS (100 ng/mL; Sigma Aldrich, St. Louis, MO, United States) and IFN-γ (50 ng/mL; Sigma Aldrich) to induce M1 polarization for a different time, or with IL-4 (25 ng/mL; Sigma-Aldrich) to induce M2 polarization for an additional 24 h.

### Delivery of AAV to the brain by stereotaxic injection

AAV administration was referred to in a previous report [24]. Briefly, prepare 2 μL per mouse of 3 × 10^12^ vector genomes of AAV in PBS. Deep anesthesia was administered to mice using isoflurane (3% to 4% for induction and 2% for 0.5% oxygen maintenance). PBS or AAV-GFP or AAV-Sbno1 was unilaterally injected with a Hamilton syringe and a 33-gauge needle at the following coordinates:+ 1.0 mm anterior/posterior (AP), ± 1.5 mm medial/lateral (ML), and − 0.8 mm dorsal/ventral from the skull (DV) for the cortex;+ 1.0 mm AP, ± 2.0 mm ML, and − 3.0 mm DV for the striatum.

### Histological analysis

Brain tissue was collected and immediately fixed in 10% paraformaldehyde. The fixed tissues were sent to Servicebio (Wuhan, China) for paraffin embedding and cut into 4-μm sections. The degree of inflammatory cell infiltration and the mean score of each sample were analyzed by two researchers according to a previous report.

### Cocl2-mediated chemical hypoxia in NSCs

The NSCs are divided into a control group and a treatment group when NSCs have good growth conditions. The treatment group cells were cultured with glucose-free deoxygenated DMEM (Thermo Fisher Scientific, Frederick, MD, USA) supplemented 200 uM CoCl2 (Sigma-Aldrich; Merck Millipore) and 100 mg/mL streptomycin and 100 U/mL penicillin in an incubator with premixed gas (95% N2 and 5% CO2) for 24 h. Cells maintained in normal media and normal conditions were used as controls.

### Isolation and identification of sEV

The sEV were isolated from the serum-free culture of NSCs. Briefly, 6 × 10^6^ NSCs were plated on a 10 cm dish and cultured in a 10 mL NSC prolifemouseion medium. After 4 days, the supernatant was collected and filtered through a 0.22 μm filter, centrifuged at 2000 g for 10 min at 4 ℃, centrifuged at 10,000 *g* for 1 h at 4 ℃, ultracentrifuged at 150,000 g for 1.5 h at 4 ℃. After centrifugation, the supernatant was removed, and the bottom of the centrifuge tube was resuspended with 100 μL PBS to precipitate and stored in the refrigerator at − 80 ℃. The sEV from the NSCs treated with AAV-GFP, AAV-Sbno1, was defined as GFP-NSC-sEV, Sbno1-NSC-sEV.

### Nanoparticle tracking analysis (NTA)

NanoSight LM10 instrument (Malvern Instruments Ltd., Malvern, United Kingdom) was used to determine the size distribution and quantity of GFP-NSC-sEV and Sbno1-NSC-sEV. The sEV samples were diluted ten times with PBS to reach optimal Concentration for instrument linearity. Data were analyzed using NTA software version 3.1.54.

### Prepamouseion of the MCAO model and evaluation

Middle Cerebral Artery Occlusion (MCAO) was performed according to a previous report. Adult C57BL/6 mice (6–8 weeks old and weighing 16–22 g) were deprived of water and grain before the 12 h of left MCAO model. After the mouse was anesthetized with isoflurane (RWD Inc., Guangzhou, China) (3% induction, 1.5% maintenance) in oxygen (0.4 L/min) and nitrogen (0.6 L/min) and routinely disinfected. The common carotid artery (CCA), external carotid artery (ECA), and internal carotid artery (ICA) were isolated and exposed fully. The embolization line was inserted into ICA via ECA; after 1 h of occlusion, the filament was removed to restore the blood flow to the MCA region. The control group was subjected to the same surgery but was not subjected to an occluded MCAO. Mice were housed in a room with a controlled temperature (22 ℃ ± 3 ℃) and humidity (60% ± 5%) under a 12-h light/12-h dark cycle. All animal experimental designs were approved by the Animal Ethics Committee of Southern Medical University, and all experiments conformed to relevant regulatory standards.

### Animal behavior test

#### Rotating-rod walking test

Place the mouse on a constant speed rotating rod of 1 rpm/min. Set to uniform acceleration mode and accelerate to 30 rpm/min for 600 s. When the mouse falls off the rotating rod, record the time, use long tweezers to promptly remove it from the device and place it back in the cage. Repeat the test two to three times at least 15 min apart.

#### Balance beam test

Mice were trained 3 days before MCAO modeling. After MCAO modeling, mice were tested on days 1, 2, 3, 5, 7, and 14, with an interval of 15 min each time. The passage time was recorded.

#### Forelimb grip strength experiment

Place the mouse on a transparent platform and hold it with its left forelimb to the tension lever. Grasp the mouse’s tail and pull it back horizontally until the left forelimb releases the lever. After the animal loses grip, record the maximum pulling force. Three times per group, with an interval of 10 min. After measuring the data of all experimental and control groups, statistical analysis was conducted.

### Protein sample preparation and western blot analysis

Total protein was extracted from cells and sEV, and the protein concentration was determined using the BCA assay as instructed. The protein samples were electrophoresed by SDS-PAGE (Sodium Dodecyl Sulfate Polyacrylamide Gel Electrophoresis) with 20 μg on the lane and then transferred onto polyvinylidene difluoride membranes for 1 h. Membranes were blocked with 5% BSA for 2 h at room tempemouseure and incubated with following antibodies overnight at 4 ℃: CD9 (1:1500, rabbit IgG; Cell Signal Technology, Danvers, MA, United States), CD81 (1:1500, rabbit IgG; Cell Signal Technology), CD63 (1:1500, rabbit IgG; Abcam, United States), TSG101 (1:1500, rabbit IgG; Abcam, United States), GAPDH (as a gel-loading control, 1:1500, rabbit IgG; Abcam, United States), Sbno1 (as a gel-loading control, 1:1500, rabbit IgG; Abcam, United States), Flag (as a gel-loading control, 1:1500, rabbit IgG; Abcam, United States). After rinsed with 1 × TBST solution, 10 min × 3 times, the membranes were then incubated with HRP conjugated sheep anti-rabbit IgG or anti-mouse IgG (1:2000, Thermo Fisher Scientific, United States) at room tempemouseure for 2 h; following rinsed with 1 × TBST, 10 min × 3 times; ECL chemiluminescence was performed to visualize the immunolabeled bands using an enhanced chemiluminescence reagent (Thermo Fisher Scientific, United States). Image J software (NIH, Bethesda, MD) was used to analyze the mean optical density of protein expression bands.

### Quantitative real-time PCR

Total RNA from cells was extracted using Trizol (Tiangen, Beijing, China). The RNA is then reversely transcribed into cDNA, reaction conditions: 37 ℃ for 60 min, 85 ℃ for 5 s. Using cDNA as a template, 2 × PCR Mix, upstream and downstream primers and sterilized double steaming water were added to a total volume of 20 μL, mixed, and put into the PCR instrument. The PCR amplification procedure was as follows: predenatumouseion at 95 ℃ for 10 min; Denatumouseion at 95 ℃ for 10 s, annealing at 60 ℃ for 20 s, extension at 72 ℃ for 15 s, 45 cycles, extension at 72 ℃ for 10 min, cooling at 25 ℃ for 30 s. Three wells were set for each sample, and GPADG was used as the internal reference primers. The primers used were synthesized by RIBBIO Biotech Company (Guangzhou, China) in this study. The relative miRNA expression of target genes was calculated by the 2^ − △△Ct^ method. Following was the primer sequence for qRT-PCR involved in this study.

### CCK8 assay

Proliferation of NSCs was assayed by the CCK8 assay (Beyotime, Shanghai, China). According to the manufacturer’s instructions, cells in 96-well plates were added with CCK8 solution and incubated for 0.5–1 h at 37 ℃. Then, the absorbance of each well was measured at 450 nm.

### Immunocytochemistry

Cells were cultured on PDL (10 μg/mL)-coated glass coverslips in 24-well plates as previously described. After the desired incubation period, cells were fixed in 4% (w/v) paraformaldehyde-sucrose for 30 min at room temperature, permeabilized with 0.2% Triton X100 and blocked for 1 h in 5% BSA. Cells were incubated with the primary antibody overnight at 4 ℃ followed by incubation with the fluorescently labeled secondary antibody for 1 h at room temperature. Primary and secondary antibodies were diluted as follows: anti-Nestin (1:500, mouse IgG1; BD Biosciences, United States), anti-SOX2 (1:500, rabbit IgG; Abcam, United States), anti-Sbno1 (1:500, rabbit IgG; Thermo Fisher Scientific, United States), goat anti-mouse IgG H&L (1:2000, Alexa fluor 647), goat anti-rabbit IgG H&L (1:2000, Alexa fluor 488). Cells were counterstained and mounted with ProLong Gold antifade reagent containing DAPI (Molecular probes, Life technologies) to visualize nuclei.

### Microscopy and image analysis

Micrographs were acquired using a fluorescence microscope (AXIO Vert.A1&Imager A2, Carl Zeiss Microscopy GmbH, Germany). ImageJ software (NIH, Bethesda, MD) randomly selected five fields under the microscope and compared five views. The number of staining positive cells in the field was calculated by taking the mean of five fields to calculate the cell density. At the same time, the average fluorescence intensity of molecular markers in brain tissue was measured by Image J software (NIH, Bethesda, MD).

### Flow cytometry analysis

The apoptosis rate was also examined by flow cytometry. After the indicated treatment, cells were harvested by centrifugation at 1100 rpm for 5 min and washed twice with PBS. The harvested cells were resuspended in fluorescein isothiocyanate (FITC)-labeled Annexin V (5 μL; BD Biosciences) and PI (5 μL; BD Biosciences) under darkness for 5 min and washed three times with PBS. Cell apoptosis rate was then estimated by flow cytometry (FACSCalibur; BD Biosciences). Data were acquired as the fraction of labeled cells or MFI within a live-cell gate using FlowJo software. All gates were set based on isotype-matched control antibodies. MAbs of mice were as follows: APC-anti-CD11b, PE -anti-F4/80, PE-cy7-anti-CD206 (BioLegend, USA).

### RNA-seq analysis

Total RNA extracted from the NSCs and Sbno1-NSCs were used for miRNA arrays. miRNA profiles were performed with ANNOROAD GENOME’s (Beijing, China) miRNA microarray service based on Affymetrix miRNA 3.0 Array. The FASTQC checked the raw fasted data quality; meanwhile, the adaptor was removed to trim quality bases by the Trimmomatic. The leading and trailing ambiguous or low-quality bases, below Phred quality scores of 3, were also removed after adapter clipping. The miRNA read counting was detected by the Chimirra, and the miRNA expressions were normalized using the trimmed mean of M-values (TMM). The edge program was further used to identify the differentially expressed genes. The gene with a fold change of expression of more than 2 was defined as a differentially expressed. The miRNA target gene prediction was detected through TargetScan (http://targetscan.org/) and miRDB (http://www.mirdb.org/). The cluster Profiler R was also performed to conduct the Gene Ontology (GO) (http://www.geneontology.org/) and Kyoto Encyclopedia of Genes and Genomes pathway (KEGG) (http://www.genome.jp/kegg/) enrichment analyses.

### Dual-luciferase reporter assay

NIH3T3 cells were co-transfected with TOP flash (a TCF reporter plasmid, Beyotime, D2503), FOP flash (which contains mutant TCF-binding sites; Beyotime, D2501) and vectors expressing either GFP or SBNO1. After 72 h, firefly and Renilla luciferase activities were measured using a Dual-Luciferase Reporter Assay System (Promega, Madison, WI, USA), according to the manufacturer’s protocol. Relative luciferase activity was reported as the firefly/Renilla luciferase activity ratio. Experiments were carried out in triplicate and repeated at least three times.

### Statistical analysis

All results were obtained from at least three independent experiments and expressed as mean ± SD. Student’s t-test was used to compare the means between two groups, while one-way ANOVA was performed for comparisons of three or more groups, followed by an LSD test for multiple comparisons. One-way ANOVA repeated measurement analysis was used to compare repeated assessments. The p < 0.05 was considered significant. Data on snRNA-seq was analyzed on the website: https://www.omicstudio.cn. Statistical analysis and graph generation were performed using SPSS Statistics 26 (Armonk, New York, U.S.), GraphPad Prism software 9 (San Diego, CA, USA), and the website https://app.biorender.com.

## Results

### Sbno1 exhibits high expression in NSCs and the central nervous system

**Fig. 1:**
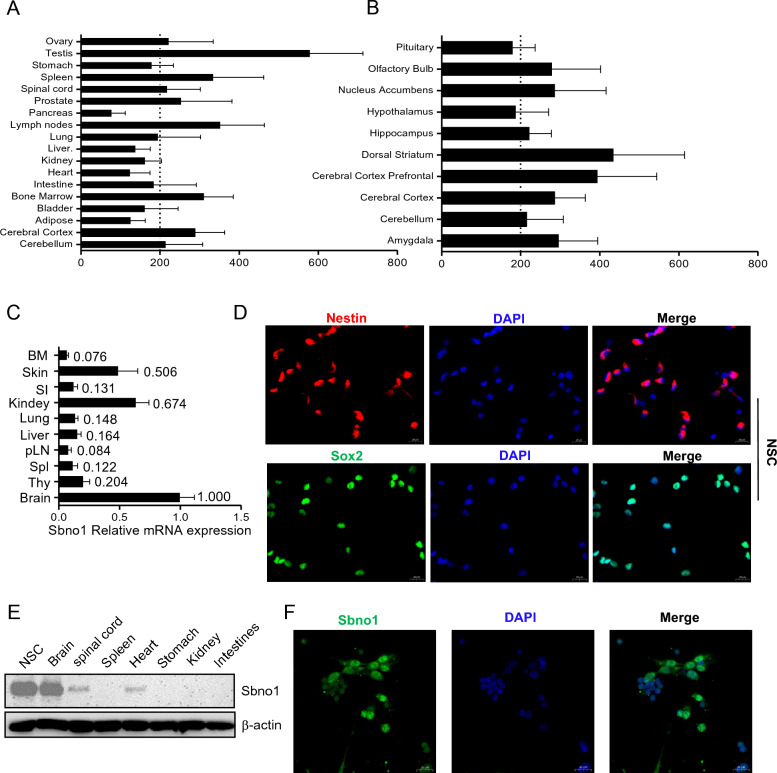
Expression and identification of Sbno1 in different tissues and organs of mice. **A**, **B** Sbno1 expression profile in various normal tissues and organs of mice as identified through the BioGPS website (http://biogps.org/#goto=welcome). **C** qRT-PCR analysis of Sbno1 expression in normal mouse organs. **D** Immunostaining of Nestin and Sox2 expression in the NSCs. Scale bar = 20 μm. **E** western blot analysis of Sbno1 expression in NSCs and normal mouse organs. **F** Immunofluorescent staining of Sbno1 in the NSCs. Scale bar = 20 μm

To investigate the expression levels of Sbno1 in tissues and organs, we analyzed the expression of Sbno1 in various normal mouse tissues and organs through the BioGPS database (Fig. 1A, B). The results revealed a higher expression level of Sbno1 in the central nervous system (CNS) than in other tissues. To validate the results from the database, qRT-PCR was performed to detect the mRNA expression levels of Sbno1 in mouse brain, spinal cord, and common visceral organs. The results showed high expression of Sbno1 in the brain (Fig. 1C). Thus, we speculated that Sbno1 may be critical in developing the nervous system. To investigate the role of Sbno1 in developing the CNS, we extracted and cultured primary NSCs and identified them through immunofluorescence [25]. The results demonstrated positive expression of Nestin and Sox2 in the primary NSCs (Fig. 1D). Furthermore, we discovered that the expression level of Sbno1 in NSCs was significantly higher than in brain tissue (Fig. 1E). Immunofluorescence staining revealed the co-localization of Sbno1 with the cell nucleus, indicating its localization as a nuclear protein (Fig. 1F). These data suggested that Sbno1 might be a potential key regulatory factor in CNS development.

### Sbno1 is positively correlated with the proliferation of NSCs

**Fig. 2:**
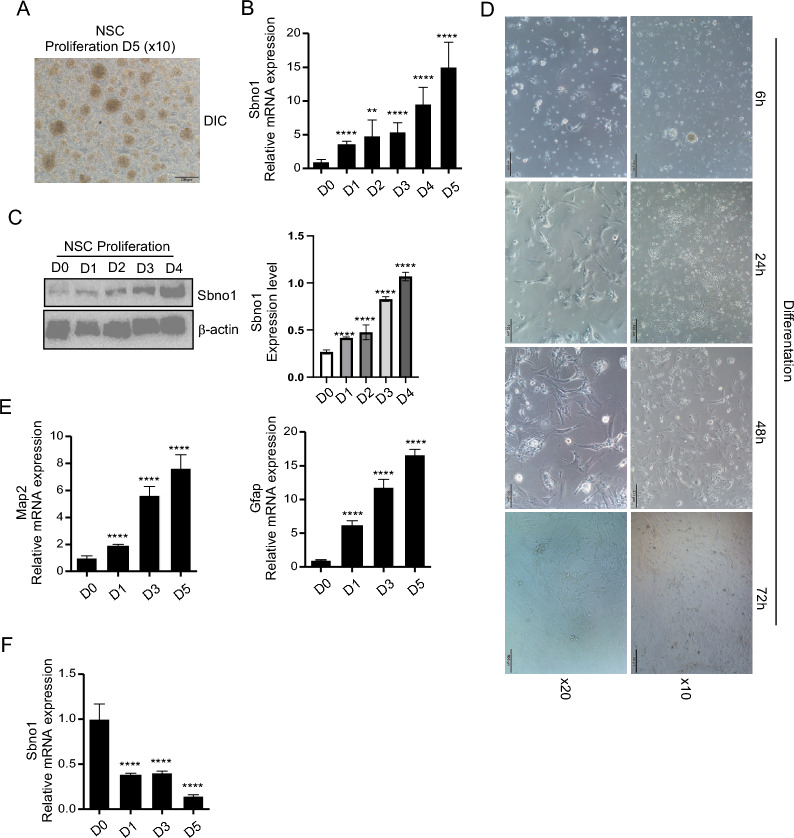
Sbno1 is specifically increased during the proliferation of NSCs. In vitro proliferative capacity of NSCs assayed by colony formation experiment. Representative image shows the neurospheres formed after 5 days of culturing. Scale bar: 200 μm. **B**, **C** qPCR and western blot analysis of Sbno1 expression during proliferation of NSCs (Full-length blots are presented in Supplementary Fig. 1). **D** Representative images show the differentiation of NSCs from 6 to 72 h after stimulated passage 1 neurospheres with differentiation medium. **E**, **F** qPCR analysis of Sbno1, Gfap and Map2 during differentiation of NSCs. **p < 0.01, ****p < 0.0001. one-way ANOVA. Data represent the mean of at least three independent experiments ± SEM

To analyze the role of Sbno1 in NSC development, we conducted expression analysis of Sbno1 at different time points during NSC proliferation and differentiation. The results revealed that the expression level of Sbno1 continuously increased during the proliferation of NSCs (Fig. 2A-C). Under differentiation culture conditions [26], we examined the cellular morphology of NSCs at the indicated time point and the mRNA expression of MAP2 and GFAP. We observed that after being stimulated with differentiation culture for 24 h, the morphology of NSCs transitioned towards neuronal and astrocytic phenotypes, completing the differentiation process within 72 h (Fig. 2D). Additionally, the expression levels of the neuronal-specific marker MAP2 [27] and the astrocytic-specific marker GFAP [28] gradually increased over time (Fig. 2E), further confirming the progressive differentiation of NSCs. Moreover, we found that the mRNA level of Sbno1 continuously decreased during NSC differentiation (Fig. 2F). In summary, our findings demonstrated a positive correlation between Sbno1 and NSC proliferation while showing a negative correlation between Sbno1 and NSC differentiation.

### Sbno1 promotes the proliferation of NSCs

**Fig. 3:**
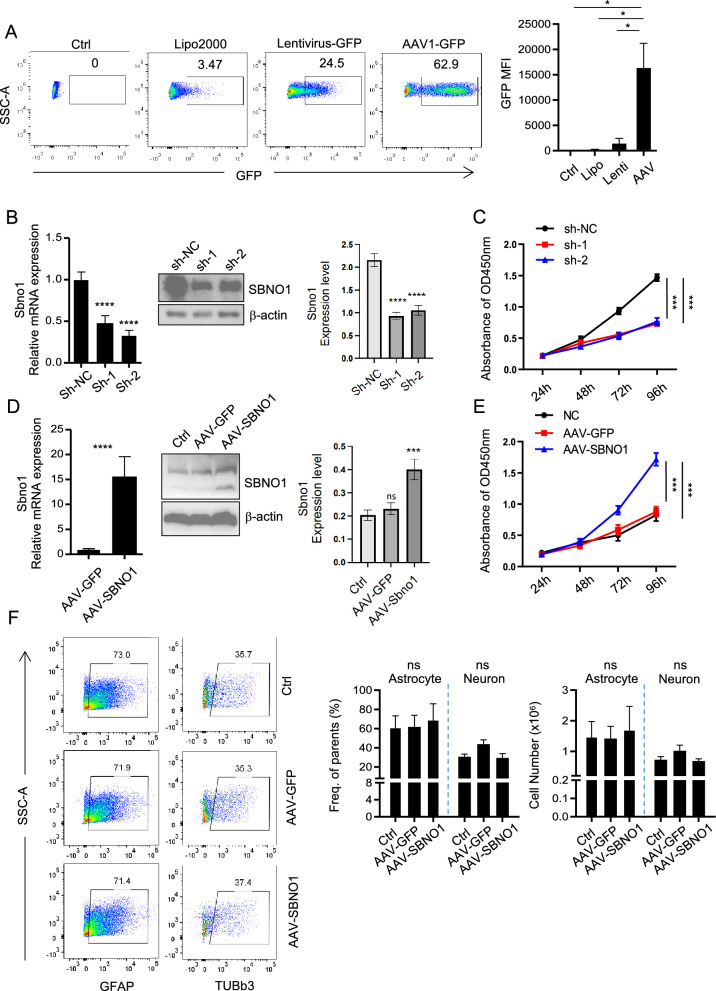
Sbno1 regulates the proliferation of NSCs. NSCs were transfected with pLVX-AcGFP1-N1 plasmid by Lipo2000 or infected with Lentivirus particles (LV-GFP) or AAV-GFP, respectively. The transfection efficiency of different methods on NSCs was detected by flow cytometry. **B** qPCR (left) and western blot (right) analysis of Sbno1 expression in the NSCs after AAV transduction of Sbno1 shRNAs or nonspecific shRNA (sh-NC).** C** Results of the cell growth curve of NSCs with Sbno1 knockdown from three independent experiments (mean ± SD). **D** qPCR (left) and western blot (right) analysis of Sbno1 expression in the NSCs infected with AAV‐expressing Sbno1 shRNAs or empty GFP vector control as indicated. **E** Results of cell growth curve of NSCs with Sbno1 overexpression from three independent experiments (mean ± SD). **F** The effect of Sbno1 overexpression on NSC differentiation. **p < 0.01, ***p < 0.001, ****p < 0.0001. (**C**, **E** one‐way ANOVA with Bonferroni correction, **D**, **F** two‐way ANOVA with Bonferroni correction)

To investigate the role of Sbno1 in the proliferation of NSCs, we leveraged AAV-mediated knockdown and overexpression of Sbno1. Previous studies have indicated that AAV exhibits high transfection efficiency in NSCs [29, 30]. We compared the transfection efficiency of liposomes, lentivirus, and AAV in NSCs and found that AAV had the highest proportion of GFP-expressing NSCs (Fig. 3A, Fig S1 A,B). Subsequently, we prepared AAV for knocking down or overexpressing Sbno1 and verified their knockdown and overexpression effects. The results demonstrated that both shRNAs effectively downregulated the Sbno1 mRNA and protein levels in NSCs, while AAV-Sbno1 effectively upregulated the expression level of Sbno1 (Fig. 3B, D). Next, we examined the changes in the proliferation capacity of NSCs after Sbno1 knockdown. We found that compared to the sh-NC group, the proliferation capacity of NSCs was significantly inhibited after Sbno1 knockdown (Fig. 3C). We also assessed the proliferation capacity of NSCs after Sbno1 overexpression and observed a significant enhancement in NSCs proliferation (Fig. 3E). These observations indicated that Sbno1 potentially facilitates the proliferation of NSCs under normal physiological conditions. Meanwhile, we also investigated the impact of Sbno1 on NSCs differentiation through flow cytometry. The results showed that overexpression of Sbno1 did not affect the differentiation of NSCs (Fig. 3F). We also unexpectedly found that Sbno1-NSCs exhibited better tolerance to CoCl_2_-induced chemical hypoxia (Fig S2 A, B).

### Sbno1 promotes the proliferation of NSCs through the Wnt/β-catenin pathway

**Fig. 4:**
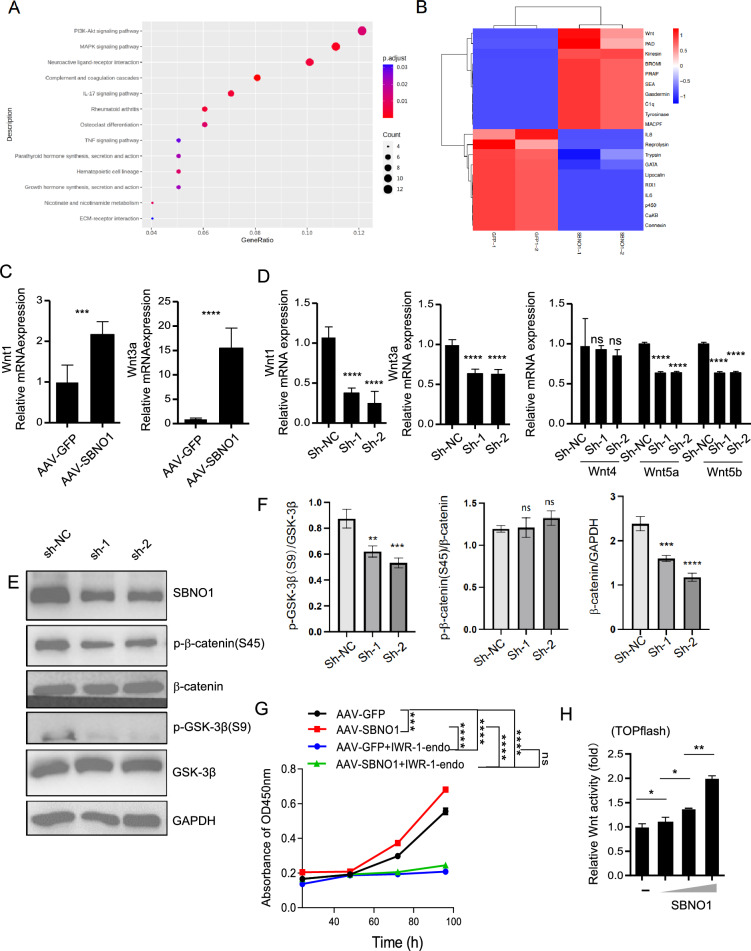
Sbno1 promotes NSC proliferation by the Wnt/β-Catenin signaling pathway. **A** KEGG pathway analysis of the RNA-seq results of Sbno1 overexpression and Control NSCs. **B** Heatmap of Sbno1 overexpression‐regulated genes compared with Control NSCs. **C** qPCR analysis of the mRNA expression of the indicated wnt/β-Catenin signaling pathway in Sbno1 overexpression and Control NSCs infected with AAV-GFP. **D-F** qPCR and western blot analysis of the mRNA and protein expression of the indicated wnt/β-Catenin signaling pathway in Sbno1 knockdown and Control NSCs infected with AAV-sh-NC. **G** Results of cell growth curve of NSCs with conditions of IWR-1-endo inhibition of the Wnt pathway from three independent experiments (mean ± SD). **H** Dual luciferase reporter assays with TOP flash, FOP flash and vectors expressing either GFP or SBNO1. **p < 0.01, ***p < 0.001, ****p < 0.0001. (**C**, **G** two‐sided Student’s t‐test, **D**, **E** one‐way ANOVA with Bonferroni correction)

We performed RNA-Seq analysis on NSCs overexpressing Sbno1, and pathway enrichment analysis revealed the most significant changes in the PI3K-Akt signaling pathway (Fig. 4A). The heatmap showed a substantial upregulation of Wnt mRNA levels in NSCs after Sbno1 overexpression (Fig. 4B). Wnt is an important signaling pathway that regulates stem cell proliferation, consisting of the Wnt canonical pathway, which plays a significant role, and the Wnt non-canonical pathway, which has a secondary role. Within the Wnt canonical pathway, β-Catenin is a crucial regulator of the Wnt signaling pathway [31–33].

To explore the signaling pathway through which Sbno1 promotes NSC proliferation, we examined the mRNA levels of Wnt signaling pathway molecules in NSCs after Sbno1 overexpression. The results showed a significant upregulation of Wnt1 and Wnt3a mRNA expression in Sbno1-overexpressed NSCs (Fig. 4C). Conversely, knocking down Sbno1 in NSCs downregulates Wnt1 and Wnt3a (Fig. 4D). Furthermore, we investigated the expression of Wnt non-canonical pathway molecules in Sbno1-knockdown NSCs and found that Sbno1 deficiency did not affect Wnt4 expression but resulted in decreased expression levels of Wnt5a and Wnt5b (Fig. 4F).

Studies have indicated that the PI3K-Akt pathway regulates the phosphorylation of GSK-3β, and phosphorylation of GSK-3β is a crucial step in activating the Wnt/β-catenin signaling pathway [34, 35]. To validate the sequencing results and further elucidate the mechanism by which Sbno1 promotes NSCs proliferation, we observed a significant decrease in the phosphorylation of GSK-3β at the S9 site after knocking down Sbno1 in NSCs (Fig. 4E). By blocking the Wnt/β-catenin pathway with IWR-1-endo [36], we found no statistical difference in the proliferation capacity between Sbno1-NSCs and GFP-NSCs after inhibition, indicating that Sbno1 primarily promotes the proliferation of NSCs through the Wnt signaling pathway. (Fig. 4G). The ability of SBNO1 to promote Wnt transcriptional activity was subsequently confirmed by transducing a dual-luciferase Wnt reporter into NIH3T3 [37]. SBNO1 overexpression significantly improved Wnt signaling in the reporter cell lines compared to the vehicle (Fig. 4H). Together, these results support SBNO1 orchestrating the proliferation of NSCs by promoting the Wnt signaling pathway during brain development. These findings revealed that Sbno1 regulates the phosphorylation of GSK-3β through the PI3K-Akt pathway, thereby influencing the activation of the Wnt/β-catenin pathway, promoting NSCs proliferation.

### Sbno1 enhances the endogenous repair capacity of NSCs

**Fig. 5:**
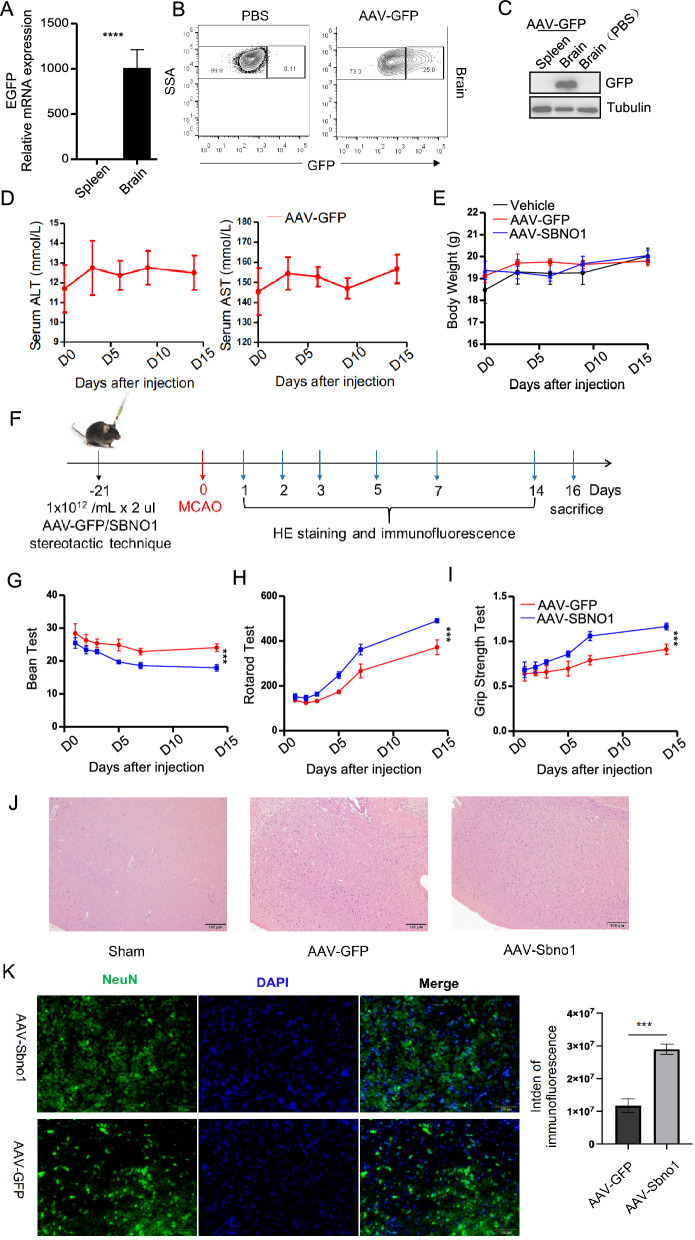
The therapeutic effect of AAV-mediated overexpression of Sbno1 in vivo on the acquired neurological impairment after ischemic stroke. **A-C** Efficiency of AAV mediated gene overexpression in vivo detected by qPCR (**A**), Flow cytometry (**B**), and western blot. **D** The impact of AAV on liver function. **E** The impact of AAV on body weight. **F** Animal experimental procedure diagram **G**–**I** Evaluation of the behavioral function of mice at 1, 2, 3, 5, 7, 14 after MCAO by balance beam test (**F**), Rotating-rod walking test (**G**), and Grip Strength Test (**H**). AAV-Sbno1 group vs AAV-GFP group. **J** Representative H&E staining of histological sections of Brain from AAV-Sbno1 group or AAV-GFP group mice. **K** Immunostaining of neurogenesis of neurons, fluorescent staining with DAPI (blue) and Neun (green). *p < 0.05, ***p < 0.001, ****p < 0.0001. (**A** two‐sided Student’s t‐test, **E**–**F**, **I** one‐way ANOVA with Bonferroni correction)

We found that the expression level of Sbno1 in neural stem cells was upregulated after stroke (Fig S4A). To further verify whether Sbno1 has potential therapeutic effects on ischemic stroke, we conducted a series of in vivo experiments. However, in vivo experiments are subject to various complex factors compared to in vitro experiments; for example, gene delivery systems’ efficiency directly impacts gene therapy’s effectiveness. We initially stereotactically injected AAV-GFP into the left hippocampal region of C57BL/6 mice, and the results demonstrated that AAV had excellent transduction efficiency in mice’s central nervous system cells (Fig. 5A, B). Furthermore, the stereotactic injection of AAV in the hippocampal region did not breach the blood–brain barrier to infect visceral organs (Fig. 5C). Simultaneously, we monitored changes in serum ALT, AST levels, and body weight. The results indicated no significant alterations in liver function or body weight following stereotactic AAV injection in the hippocampal region (Fig. 5D, E). Mice with a body weight between 18 and 20 g were randomly divided into two groups. Animal models were constructed by stereo tactically injecting AAV-Sbno1 or AAV-GFP into the brain. Subsequently, cerebral artery occlusion was induced in both groups to create a model of middle cerebral artery stroke. Five mice with a Longa score of 2 from both groups were selected for the balance beam test, rotarod test, and assessment of grip strength in the right forelimb to evaluate their post-stroke prognosis (Fig. 5F). The results showed that the recovery of balance, limb coordination, physical stamina, and strength in mice treated with AAV-Sbno1 was superior to the AAV-GFP group (Fig. 5G-I). Furthermore, we conducted HE and immunofluorescence staining to examine the post-stroke brain’s pathological conditions and explore the underlying reasons for the enhanced post-stroke recovery in mice with hippocampal overexpression of Sbno1. The results showed that in the acute phase of stroke (72 h), there is a lower level of inflammatory cell infiltration in Sbno1-overexpressed mice compared with control mice (Fig. 5J). Neurons, the most crucial functional cells in the central nervous system, do not possess inherent self-replication capabilities. Each neuron can only originate from the differentiation of a neural stem cell. Hence, the quantity of NSCs directly influences the number of newly generated neurons. In the recovery phase of stroke (28 days), mice subjected to AAV-Sbno1 exhibited more neurons than the control group (Fig. 5K). These data demonstrated that enhancing the proliferative capacity of NSCs can increase the population of newly formed neurons, thereby promoting endogenous neural repair.

### Sbno1-NSCs-sEV suppressing neuroinflammation by inhibiting the MAPK and NF-κB signaling pathways

**Fig. 6:**
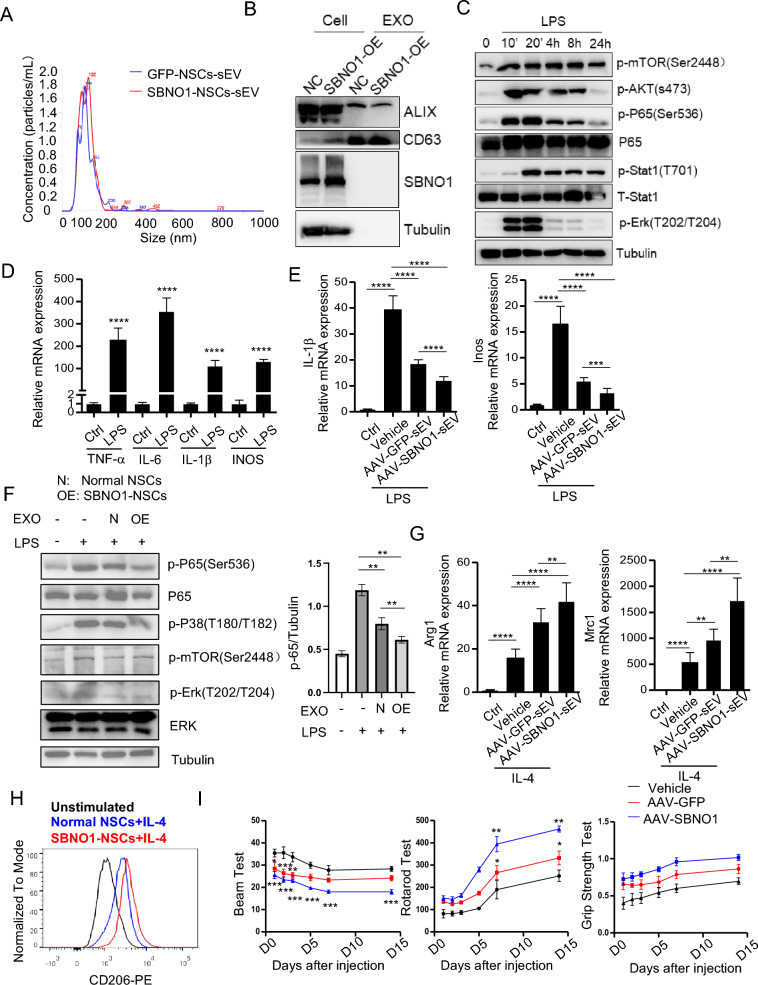
Sbno1-NSCs-sEV inhibits the development of neuroinflammation after stroke by inhibiting M2 polarization of microglia. An NTA analysis indicated a similar size range for NSCs-sEV(2.68 × 10^11^ p/mL) and Sbno1-NSCs-sEV(1.23 × 10^11^ p/mL). **B** Western blot analysis was conducted to detect the expression of specific extracellular vesicle-related positive markers (CD63, Alix) and Sbno1 in Sbno1-NSCs-sEV and NSCs-sEV. **C** Western blot analysis of the activation of key molecules in downstream signaling pathway after LPS induced M1 polarization of microglia cells. **D** qPCR analysis of the mRNA expression of LPS induced M1 type inflammatory factor expression in microglia cells. **E** qPCR analysis of the IL-1β(left) and Inos (right) mRNA expression of Sbno1-NSCs-sEV or NSCs-sEV pretreated microglia cells after LPS stimulation. **F** Western blot analysis of the activation of the NF-κB, mTOR, MAPK pathway by LPS in microglia cells pretreated with Sbno1-NSCs-sEV or NSCs-sEV. **G** qPCR analysis of the Arg1(left) and Mrc1 (right) mRNA expression of Sbno1-NSCs-sEV or NSCs-sEV pretreated microglia cells after IL4 stimulation. **H** Overlaid histograms show Mrc1 levels in M2 microglia pretreated with Sbno1-NSCs-sEV or NSCs-sEV.** I** Evaluation of the behavioral function of mice at 1, 2, 3, 5, 7, and 14 after MCAO by balance beam test, Rotating-rod walking test, and Grip Strength Test in Vehicle group or Sbno1-NSCs-sEV group or NSCs-sEV group. *p < 0.05, **p < 0.01, ***p < 0.001, ****p < 0.0001. (**D**, **E**, **G**) two‐sided Student’s t‐test, **I** two‐way ANOVA with Bonferroni correction)

NSCs could reduce oxidative stress and inflammation in the brain by secreting sEV. Microglial mediate the occurrence of neuroinflammation after ischemic stroke [38–40]. It has suggested the involvement of sEV in regulating inflammation [41]. Therefore, further investigate the reasons for the lower infiltration of inflammatory cells in the hippocampus of mice overexpressing Sbno1 after stroke, we conducted NTA and WB to identify sEV derived from NSCs (Fig. 6A, B). The results revealed that Sbno1-NSCs-sEV, secreted by NSCs overexpressing Sbno1, exhibited similar diameter and surface molecular markers to the control group. Furthermore, Sbno1 was not found to be carried and secreted in the form of a protein by sEV into the extracellular environment. We discovered that Sbno1-NSCs-sEV could modestly upregulate the expression of M2 phenotype molecules Arg1 and Mrc1 in macrophages (Fig S3A-B). To investigate whether Sbno1-NSCs-sEV regulates the occurrence of neuroinflammation, we mimicked the process of neuroinflammation by inducing M1 polarization in microglial using lipopolysaccharide (LPS) (Fig. 6C, D). We found that pretreatment with Sbno1-NSCs-sEV, compared to GFP-NSCs-sEV, could suppress the expression of IL-1β and Inos induced by LPS in microglial (Fig. 6E) at the same concentration.

To further investigate the mechanical effects of Sbno1-NSCs-sEV in suppressing neuroinflammation, we examined the expression level of Sbno1 and the alterations of NF-κB, mTOR, and MAPK/ERK signaling pathways during M1 polarization of microglial. The results showed that during the process of ischemic stroke, with the M1 polarization of microglia and neuroinflammation, the number of cells double-positive for Sbno1 and the microglial marker IBA-1 continued to increase (Fig S3C). Meanwhile, compared to GFP-NSCs-sEV, Sbno1-NSCs-sEV significantly enhanced the inhibition of microglial activation, particularly phosphorylation of P38 and P65, thereby suppressing their polarization towards the M1 phenotype (Fig. 6F). Furthermore, we assessed the expression of M2 markers (Arg1 and Mrc1) during the M2 polarization of the microglial. We found that Sbno1-NSCs-sEV upregulated the expression of Arg1 and Mrc1 following IL-4 stimulation, thereby promoting microglial polarization to the M2 phenotype and exerting anti-inflammatory effects (Fig. 6G, H). We also found that the anti-inflammatory effects of Sbno1 were significantly attenuated when the secretion of NSCs-sEV was blocked. This result suggests that sEV is an essential pathway for Sbno1’s involvement in regulating neuroinflammation (Fig S3D, E). To further validate the inhibitory effect of Sbno1-NSCs-sEV on neuroinflammation in vivo, we stereotactically injected Sbno1-NSCs-sEV, GFP-NSCs-sEV, or PBS into the ischemic penumbra region of the mouse brain after MCAO with Longa score of 2. The results demonstrated that mice treated with Sbno1-NSCs-sEV exhibited the most significant recovery in performance, as assessed by the balance beam test, rotarod test, and grip strength evaluation of the right forelimb (Fig. 6I).

### AAV-Sbno1 possesses multiple biological effects, and administering gene therapy to stroke patients early can help improve their prognosis

**Fig. 7:**
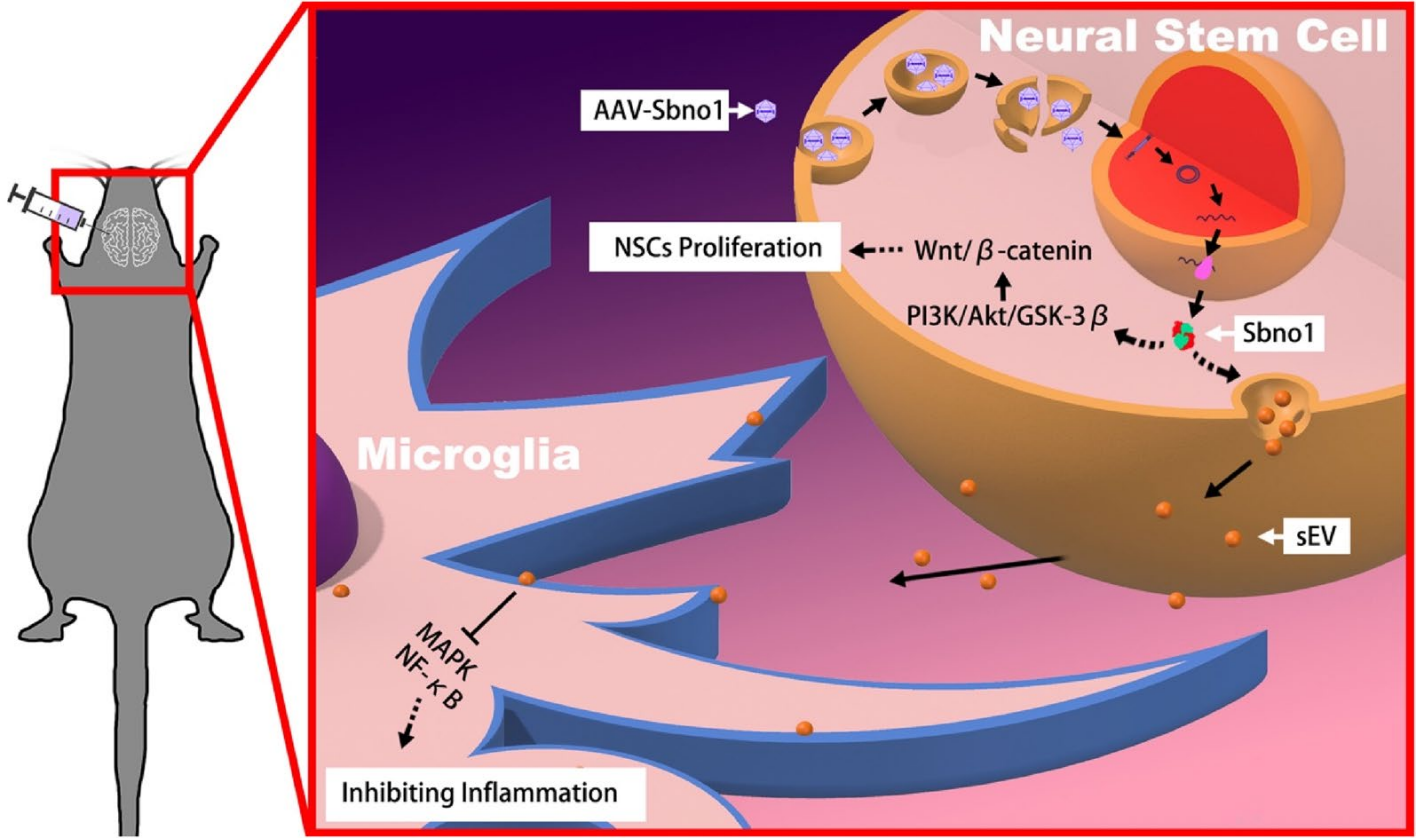
Model Diagram of AAV-Sbno1 Gene Therapy Mechanism. Sbno1 enhances the phosphorylation of GSK-3β through the PI3K-Akt signaling pathway, thereby upregulating the Wnt/β-catenin signaling pathway and promoting the proliferation of NSCs. At the same time, Sbno1 regulates the secretion of NSCs sEV and inhibits the NF-κB and MAPK signaling pathways in microglia, thereby suppressing M1 polarization of microglial. Under the combined action of these two biological effects, Sbno1 promotes endogenous repair and inhibits neuroinflammation

Based on the data presented earlier, AAV-Sbno1 exhibits a dual effect by suppressing neuroinflammation and enhancing endogenous stem cell repair. Early administration of AAV-Sbno1 to stroke mice could inhibit the further worsening of the disease caused by neuroinflammation and improve prognosis by enhancing the endogenous repair capabilities of neural stem cells (Fig. 7). Therefore, AAV-Sbno1 gene therapy possesses a dual therapeutic effect, making it a potential treatment option for acute-phase stroke patients.

## Discussion

Stroke ranks as the second leading cause of death globally, the third leading cause of disability, and a significant contributor to cognitive decline and dementia [42–44]. Currently, clinical treatment for ischemic stroke primarily focuses on thrombolysis. However, the narrow time window for thrombolysis results in many patients not receiving timely treatment, leading to various degrees of motor dysfunction. Neurons, as permanent cells, cannot self-replicate and self-repair. In contrast, NSCs, as essential stem cells, possess the potential for self-renewal and differentiation into neurons, offering the potential to improve the prognosis of stroke patients.

The adult hippocampus harbors naturally occurring NSCs in a quiescent state [45]. These endogenous NSCs within the central nervous system present the optimal choice for intrinsic repair due to their innate biological safety. However, the quantity of endogenous NSCs is minimal, and their inherent repair capacity is insufficient. Relying solely on their capabilities, they cannot fully restore the limb disabilities caused by stroke. To harness their therapeutic potential, the primary challenge lies in promoting the self-amplification of these rare NSCs to facilitate their intrinsic reparative function. The Wnt signaling pathway serves as a crucial regulatory pathway governing the proliferation of various stem cells. Enrichment analysis of RNA-Seq data from NSCs overexpressing Sbno1 reveals a significant PI3K/Akt pathway activation. Activation of the PI3K/Akt pathway facilitates the phosphorylation of GSK-3β at Ser9 and then activates the Wnt/β-catenin signaling pathway [46], directly influencing the nuclear translocation of β-catenin and promoting stem cell proliferation. With the recent FDA approval of two AAV drugs for treating rare genetic diseases, AAV vectors are now on the market and are being further explored for other therapies [46]. For patients with disabilities following a stroke, gene therapy utilizing AAV carrying Sbno1 can upregulate the expression of Sbno1 in human hippocampal NSCs, thereby promoting the proliferation of endogenous NSCs and intrinsic repair. AAV vectors do not integrate the target gene into the genome, showing excellent biosecurity [47–49]. Clinical practitioners can flexibly decide whether to perform secondary or multiple gene therapies based on the patient’s recovery status after the initial AAV-Sbno1 gene therapy. Our animal experiments did not observe tumorigenic or teratogenic effects from AAV-Sbno1 gene therapy, nor did they harm liver or kidney function. Therefore, we believe that gene therapy based on AAV-Sbno1 holds immense clinical research value for improving the prognosis and enhancing the quality of life for stroke patients.

Exogenous NSC transplantation has been extensively studied in the field of central nervous system diseases [50–55]. Particularly in neurodegenerative diseases, it has demonstrated efficacy in partially restoring neural function in experimental settings [56–58]. However, unlike the initial success observed in stem cell therapies for other central nervous system diseases, there are still numerous unresolved issues between NSC transplantation and clinical treatment for acute ischemic stroke [59]. The process of exogenously transplanted NSCs differentiating into neurons and glial cells within the body is akin to the growth of a seed into a towering tree. It requires robust seeds, fertile soil, and a relatively stable external environment. However, NSCs have high metabolic demands and poor tolerance, and even in vitro culture necessitates strict conditions. Obviously, NSC is not a potent seed. After transplantation into the area of stroke injury, it is challenging for NSCs to survive in the pathological environment characterized by inflammatory cell infiltration, ischemia, and hypoxia. This intricate extracellular environment is not the fertile soil conducive to seed germination. Therefore, in this pathological condition of ischemia and hypoxia, we have chosen our research direction to focus on controlling the inflammatory storm and activating and enhancing the individual’s intrinsic repair capabilities.

The vascular system in the brain is complex. After ischemic stroke occurs, in addition to the irreversible death of neurons in the ischemic core region, the microglial attack damaged neurons in the surrounding ischemic penumbra, leading to an expansion of the injury area and exacerbation of the damage. Therefore, providing necessary anti-inflammatory treatment can protect the damaged neuronal cells in the ischemic penumbra. However, due to the obstruction caused by cerebral vascular embolism and the blood–brain barrier, there are difficulties in drug delivery [60]. The anti-inflammatory effect exhibited by Sbno1 undoubtedly provides a solution to this issue. AAV-Sbno1 gene therapy can achieve both the activation of NSCs and the suppression of neuroinflammation with a single intracranial injection.

The contents of exosomes include various RNAs that can collectively regulate the polarization process of inflammatory cells through multiple signaling pathways [41, 61, 62].We have discovered that Sbno1, in the form of sEV, participates in the signaling communication between NSCs and microglial. It can inhibit the NF-κB and MAPK signaling pathways in microglial and suppress M1 polarization of microglial. Furthermore, it can upregulate the expression of MRC1, promoting M2 polarization of microglial and inhibiting neuroinflammation. Subsequently, through histological examination (HE and immunohistochemical staining), we confirmed that overexpressing of Sbno1 in the hippocampal region exhibited lower levels of neuroinflammation and a higher number of surviving neurons following ischemic stroke. To further investigate the therapeutic effects of sEV, we administered Sbno1-NSCs-sEV, GFP-NSCs-sEV, or PBS (control) via stereotactic intracerebral injection to mice after stroke. On day 7, the Sbno1-NSCs-sEV treatment group exhibited improved behavioral scores. SEV serve as excellent drug carriers capable of crossing the blood–brain barrier [63]. Studies have shown that the combination therapy of sEV with tPA thrombolysis demonstrates promising treatment outcomes [64]. Moreover, Sbno1-NSCs-sEV possess a more vital ability to suppress neuroinflammation, offering better patient central nervous system protection and reducing inflammatory damage. They also hold the potential for combined therapy with tPA thrombolysis. This suggests that gene therapy based on AAV-Sbno1 can not only treat the physical disabilities caused by stroke but also has therapeutic effects on the neuroinflammation in the acute phase of stroke. It further underscores the importance of early administration of AAV-Sbno1 gene therapy for stroke patients to reduce secondary damage caused by stroke-induced neuroinflammation. However, this study has some limitations. First, a further in-depth research is needed to elucidate the signaling pathways through which Sbno1 regulates the proliferation of NSCs. Second, due to the extended duration required for AAV-Sbno1 overexpression and the relatively rapid recovery of mice with Longa scores of 2 points after MCAO, we could only generate mice with Sbno1 overexpression first and then induce MCAO, which doesn’t fully replicate the disease onset and treatment process of AAV-Sbno1 gene therapy post-MCAO. Third, although we have discovered that sEV secreted by Sbno1-NSCs have anti-inflammatory effects on microglial, further investigation is needed to determine the specific changes in sEV contents that contribute to their enhanced anti-inflammatory properties. Gene therapy with AAV-Sbno1 can be tailored to the patient’s disease progression, allowing for flexible dosing frequency and dosage determination, thus achieving personalized medicine. It also shows potential in combination with tPA thrombolysis therapy. These findings offer new avenues for gene therapy for stroke and improving the prognosis of stroke patients.

## Conclusion

In summary, our results indicated that Sbno1 activated the Wnt/β-catenin pathway, promoting the proliferation of NSCs and enhancing their endogenous repair effects. Sbno1 also enhanced the tolerance of NSCs to ischemia and hypoxia, thereby improving their survival rate after stroke. Moreover, Sbno1, in the form of sEV secreted by NSCs, could inhibit the NF-κB and MAPK signaling pathways in microglial, suppress M1 polarization and promote microglial polarized to M2 subtype.

## Supplementary Information

Additional file 1: Fig. 1 AAV-GFP Infected NSCs. A， B Fluorescence imaging of neurospheres after AAV administration for 48 h. Supplementary Fig. 2 Sbno1 could Inhibit Apoptosis of NSCs. A， B Flow cytometry analysis of the percentages of cell death in NSCs induced chemical hypoxia by Cocl2. Fig. 3 Sbno1 could inhibit the neuroinflammation. A， B qPCR analysis of the mRNA expression of LPS induced M1/2 type inflammatory factor expression in microglia cells. C Immunofluorescence staining of Sbno1 in microglial after ischemic stroke. D Western blot analysis of the activation of the p-P65 by LPS in microglia cells pretreated with Sbno1-NSCs-sEV /NSCs-sEV or GW4869. E qPCR analysis of the IL-6(left) and TNF-α (right) mRNA expression in in microglia cells which treated with NSCs-CM or NSCs-CM pretreated with GW4869 or Sbno1-NSCs-sEV followed with LPS stimulated. (CM= Conditional medium; GW4869: exosome inhibitor). Fig. 4 Immunofluorescence staining of Sbno1 in NSCs， neurons， microglial, and astrocytes after ischemic stroke

## Data Availability

The data supporting this study’s findings are available from the corresponding author upon reasonable request.

## Abbreviations

AAV: Adeno-associated virus
GFAP: Glial Fibrillary Acidic Protein
GSK-3β: Glycogen Synthase Kinase-3 beta
iPSCs: Induced pluripotent stem cells
LPS: Lipopolysaccharide
MAP2: Microtubule-Associated Protein 2
MAPK: Mitogen-Activated Protein Kinase
MCAO: Middle Cerebral Artery Occlusion
NF-κB: Nuclear Factor-kappa B
NSCs: Neural stem cells
NTA: Nanoparticle tracking analysis
Sbno1: Strawberry Notch 1
sEV: Small extracellular vesicle
