# Supplementary figures and images for: Sbno1 mediates cell–cell communication between neural stem cells and microglia through small extracellular vesicles
Source: Cell Biosci. 2024 Sep 29;14:125. doi: 10.1186/s13578-024-01296-4 (PMC11441009; doi:10.1186/s13578-024-01296-4)

Figure 1E

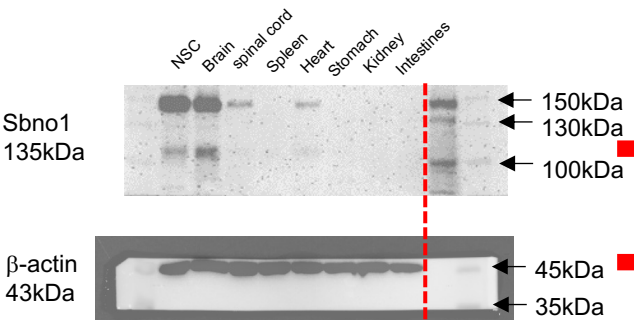

Figure 1E

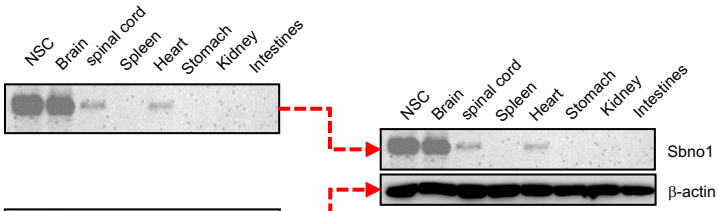

Figure 2C

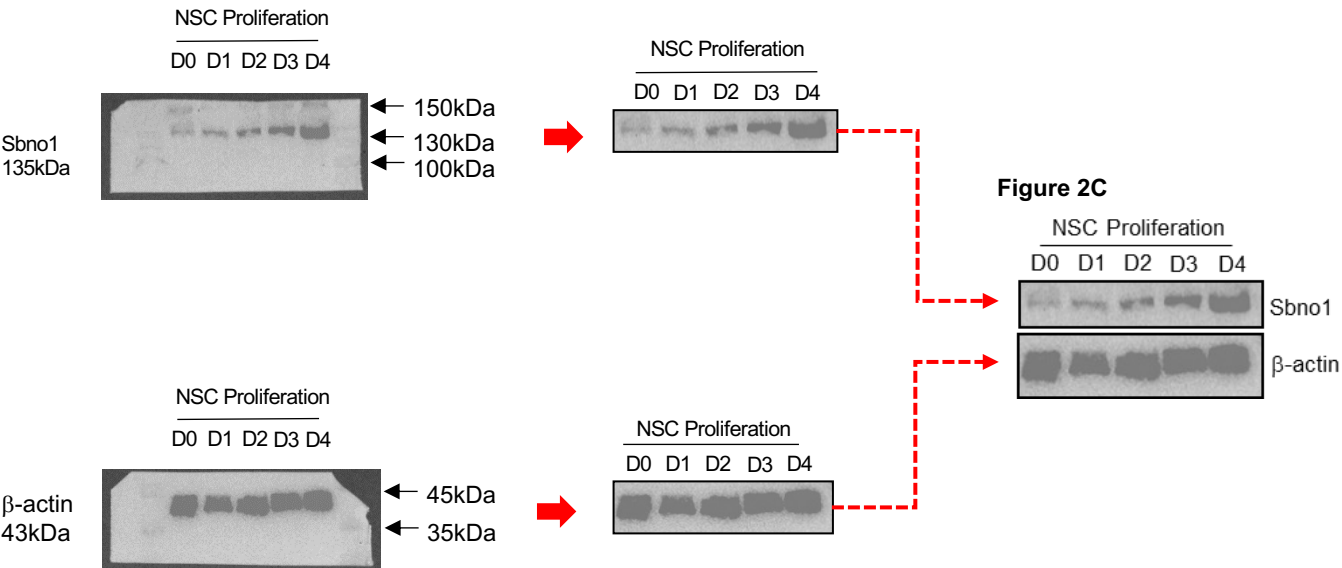

Figure 3B

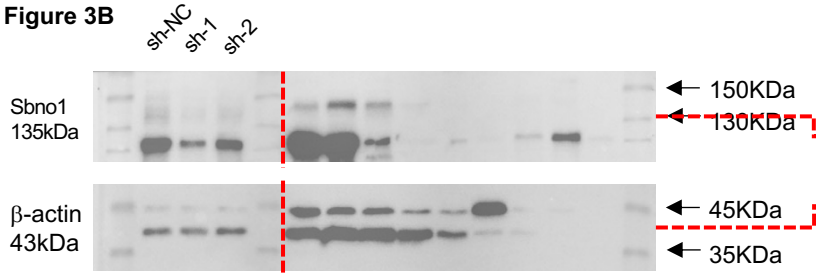

Figure 3B

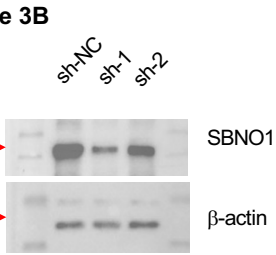

Figure 3D

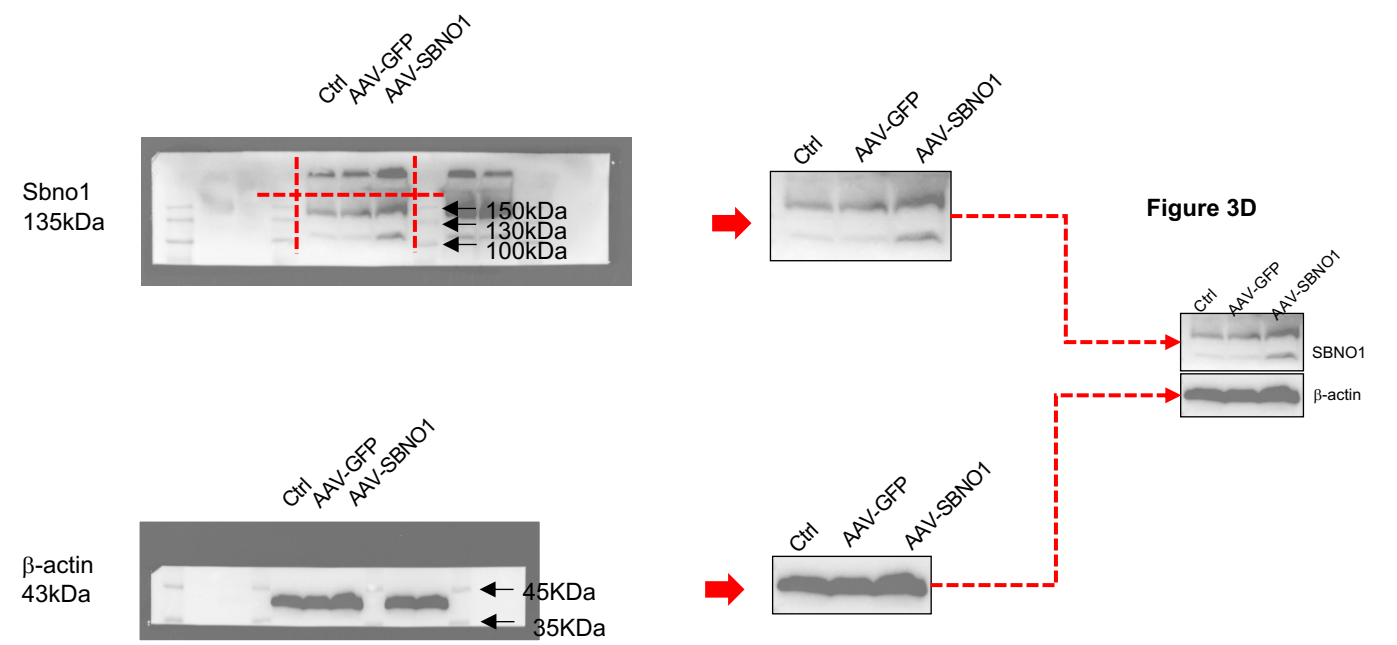

Figure 4E

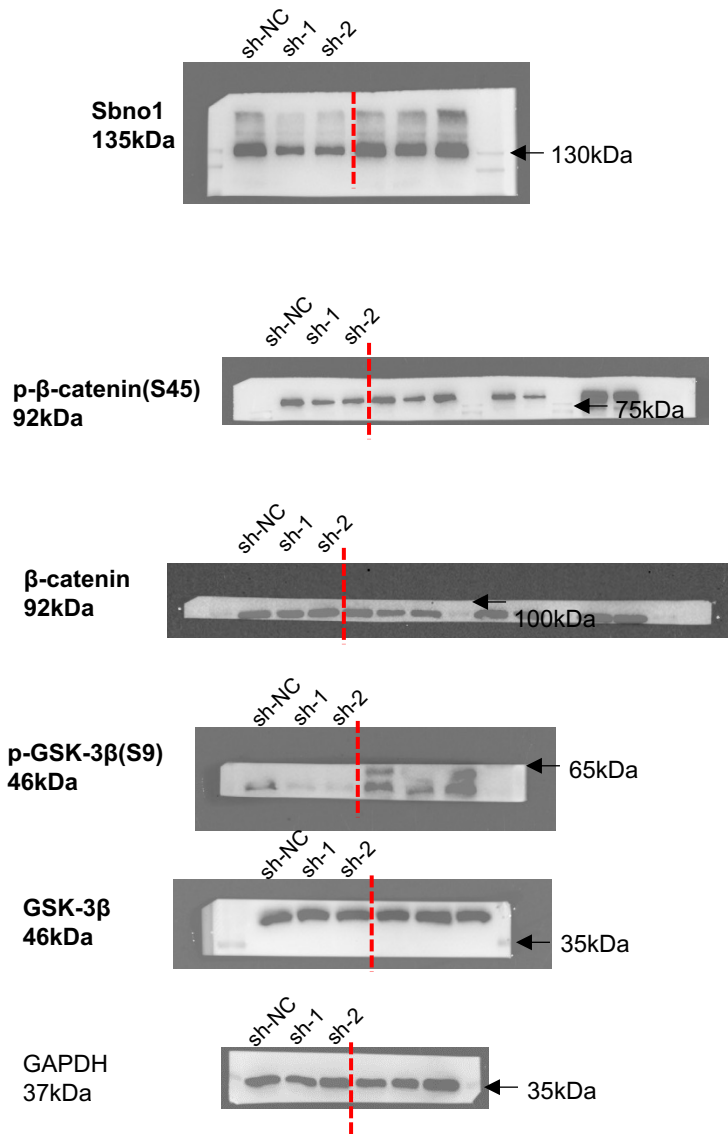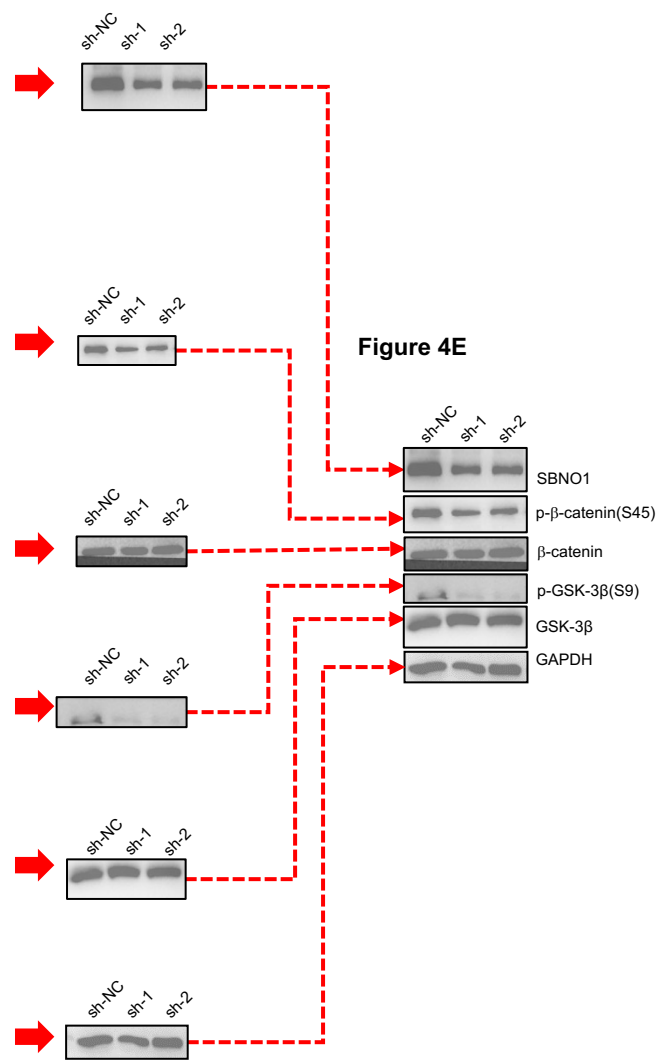

Figure 5C

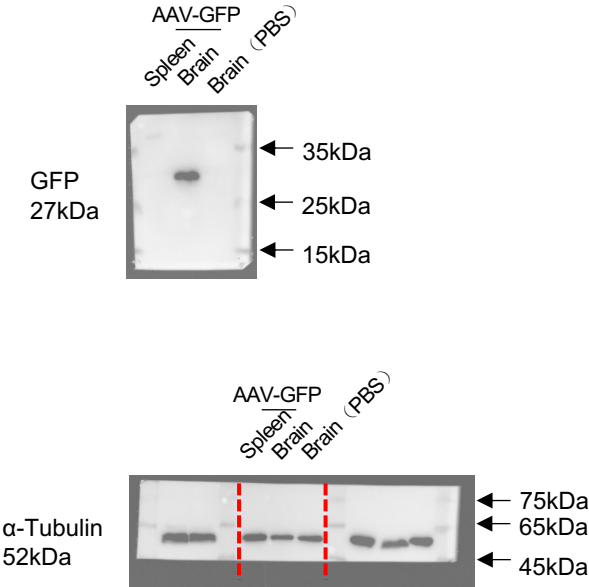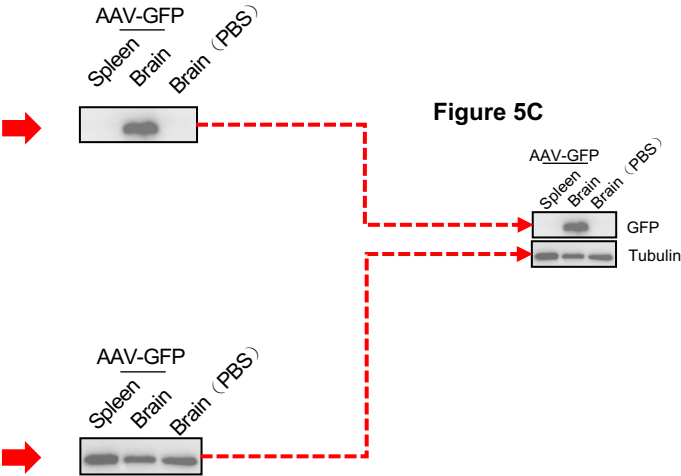

Figure 6B

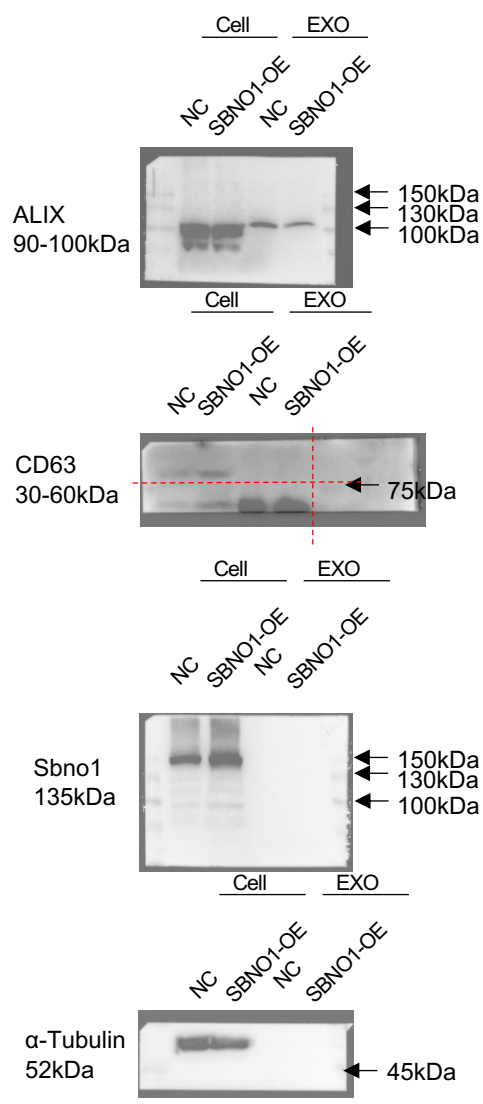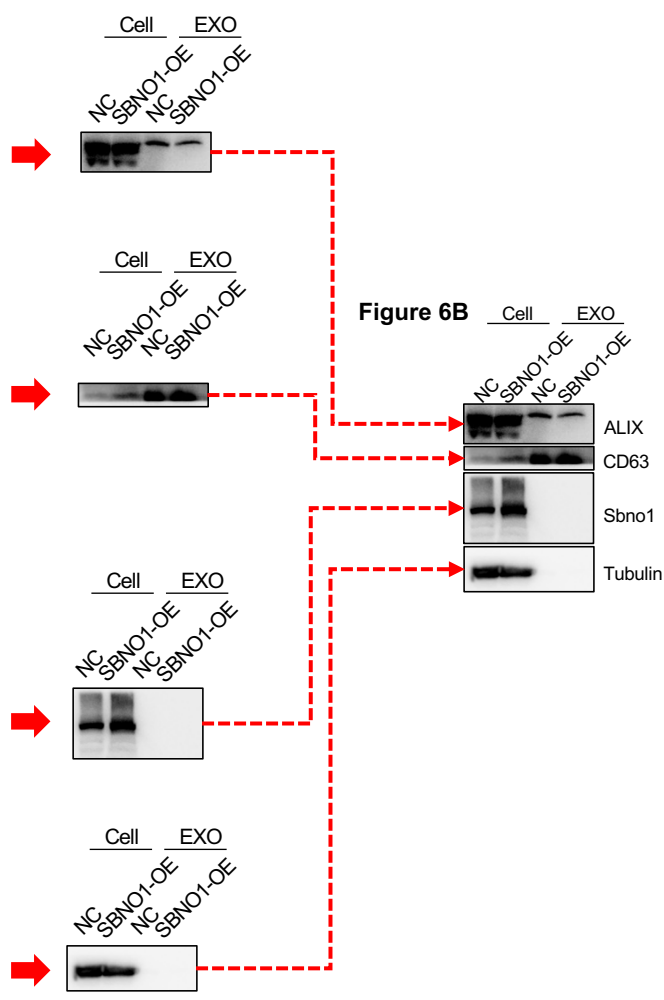

Figure 6C

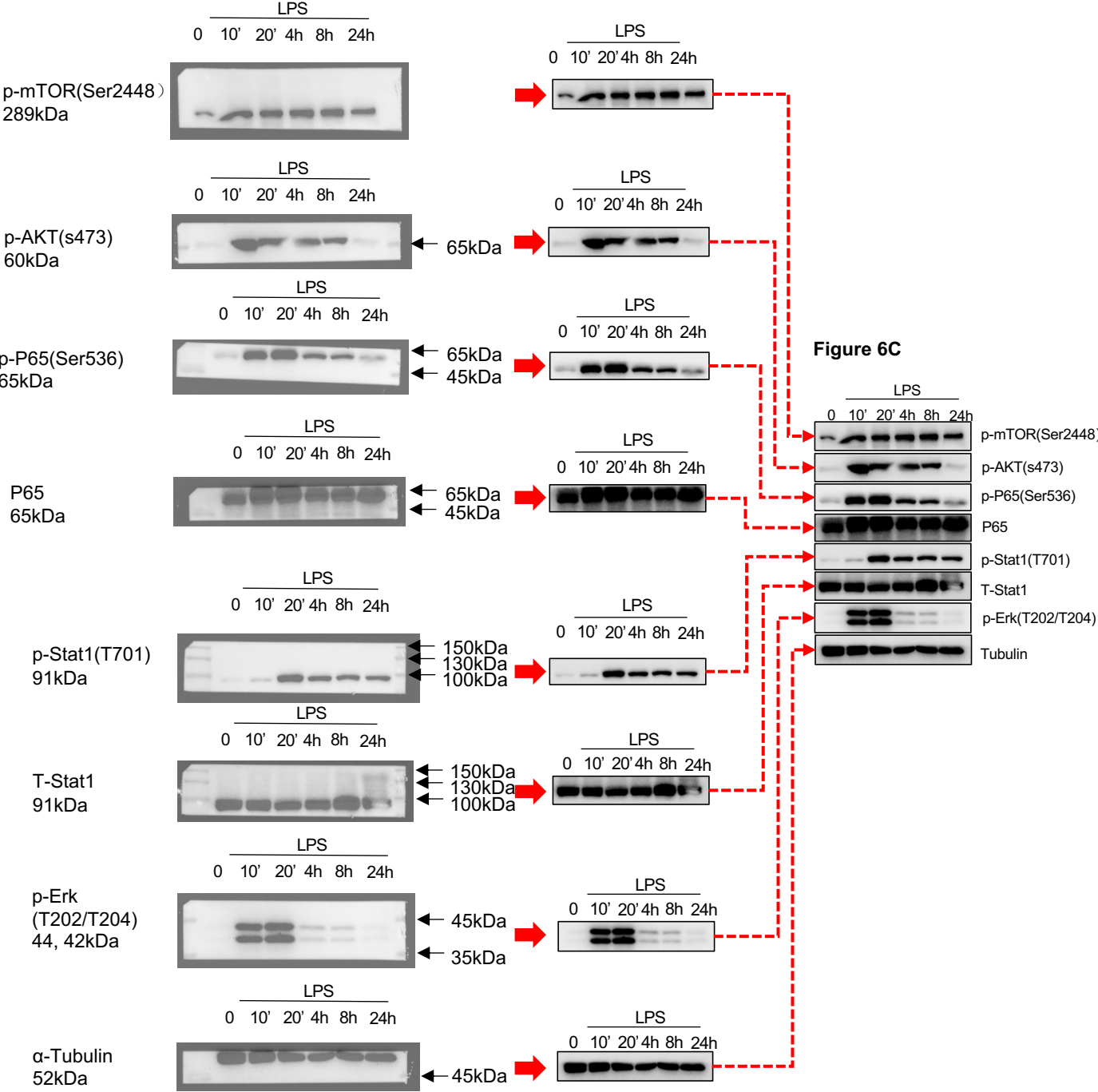

Figure 6F

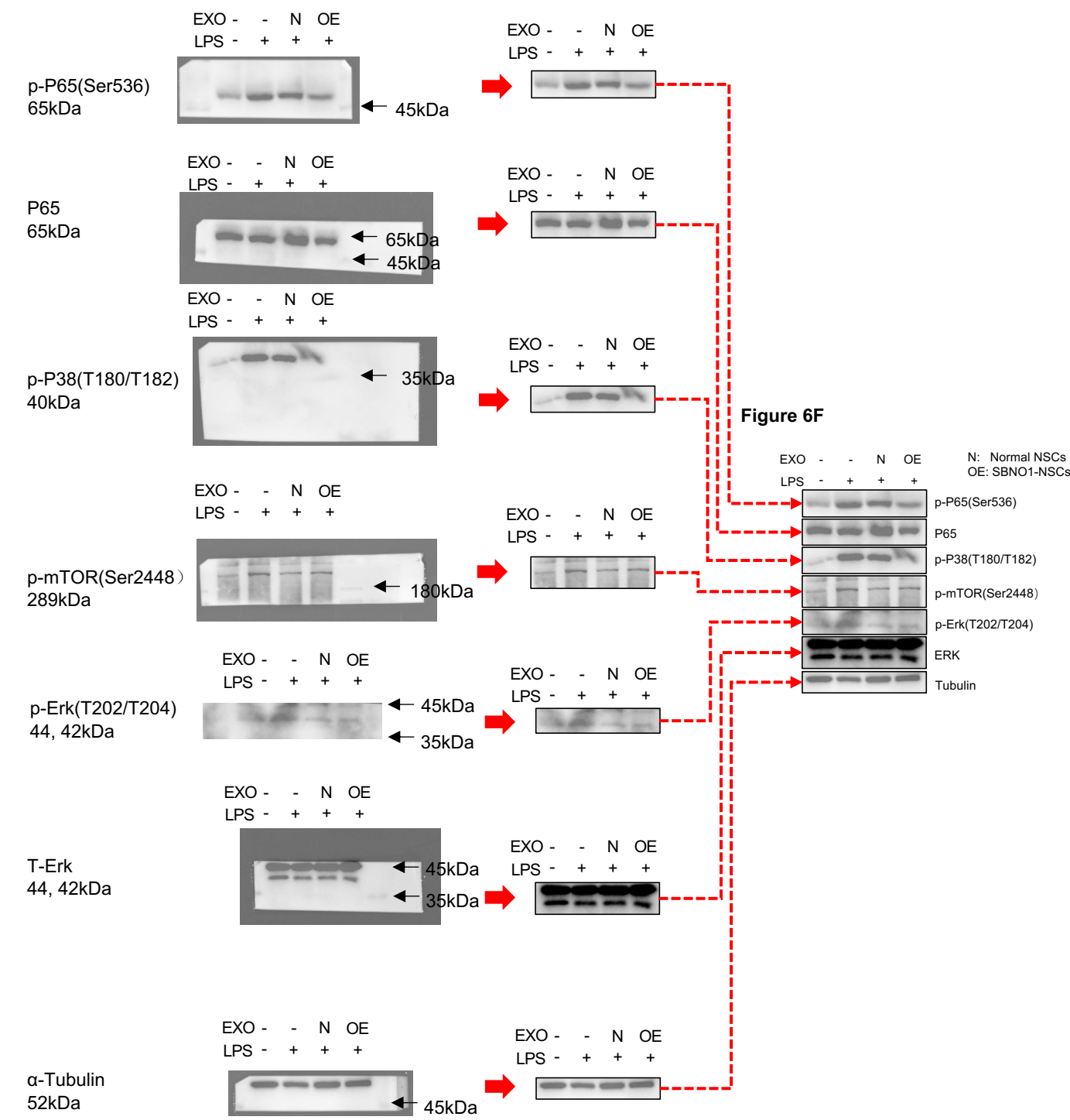

Supplement: Supplementary file 1 — Additional file 1: Fig. 1 AAV-GFP Infected NSCs. A， B Fluorescence imaging of neurospheres after AAV administration for 48 h. Supplementary Fig. 2 Sbno1 could Inhibit Apoptosis of NSCs. A， B Flow cytometry analysis of the percentages of cell death in NSCs induced chemical hypoxia by Cocl2. Fig. 3 Sbno1 could inhibit the neuroinflammation. A， B qPCR analysis of the mRNA expression of LPS induced M1/2 type inflammatory factor expression in microglia cells. C Immunofluorescence staining of Sbno1 in microglial after ischemic stroke. D Western blot analysis of the activation of the p-P65 by LPS in microglia cells pretreated with Sbno1-NSCs-sEV /NSCs-sEV or GW4869. E qPCR analysis of the IL-6(left) and TNF-α (right) mRNA expression in in microglia cells which treated with NSCs-CM or NSCs-CM pretreated with GW4869 or Sbno1-NSCs-sEV followed with LPS stimulated. (CM= Conditional medium; GW4869: exosome inhibitor). Fig. 4 Immunofluorescence staining of Sbno1 in NSCs， neurons， microglial, and astrocytes after ischemic stroke [file 13578_2024_1296_MOESM1_ESM.pdf]
